# Sustainable Approaches of Plant Nutrient and Environment Management to Plant Production

**DOI:** 10.3390/plants15010032

**Published:** 2025-12-22

**Authors:** Zaid Khan, Mohammad Nauman Khan, Junguo Bi, Lijuan Xie

**Affiliations:** 1 College of Architectural Engineering, Shenzhen Polytechnic University, Shenzhen 518055, China; zaidkhan@scau.edu.cn; 2 College of Natural Resources and Environment, South China Agricultural University, Guangzhou 510642, China; 3 School of Breeding and Multiplication (Sanya Institute of Breeding and Multiplication), Hainan University, Sanya 572025, China; naumanagrarian@yahoo.com; 4 Shanghai Agrobiological Gene Center, No. 2901, Beidi Road, Shanghai 201106, China; gbi@sagc.org.cn

## 1. Introduction

The adoption of sustainable plant production techniques is crucial to addressing the issues of food security, environmental degradation, and climate change [1]. Increasing crop output while minimizing ecological impacts depends critically on the effective management of plant nutrients and the surrounding environment [2]. In modern agriculture, striking a balance between the optimal use of nutrients and ecological preservation remains a significant challenge. The goal of this Special Issue (SI) is to investigate sustainable methods for managing plant nutrients and the environment, with an emphasis on creative tactics that not only increase plant productivity and growth but also support soil health, reduce environmental pollution, and enhance resistance to changing climate conditions.

This Special Issue’s study provides a comprehensive strategy for dealing with these issues. It compiles research on a wide range of plant species and agricultural practices, from wetland vegetation to rice, soybeans, and litchi. The contributions span a wide range of geographical areas, including Asia, Europe, and the Americas, and provide a comprehensive understanding of sustainable plant production methods. To promote sustainability in plant production, topics such as nutrient management techniques, the role of external inputs, soil amendments, and environmentally friendly fertilization systems are examined. This Special Issue is a valuable resource for scholars and practitioners seeking more productive and sustainable agricultural systems, as it provides both theoretical foundations and practical solutions.

Maintaining the long-term health of agricultural ecosystems while increasing resource efficiency requires sustainable nutrient management. Optimizing fertilizer application has been the focus of recent research aimed at reducing nutrient leaching, increasing nutrient uptake, and enhancing soil health [2]. To reduce reliance on artificial fertilizers and improve environmental sustainability, technologies such as organic amendments and biotechnological interventions, including the use of microorganisms that promote plant growth, are being increasingly employed [3]. Furthermore, to sustain productivity in the face of shifting climate conditions, efforts to mitigate abiotic stresses, including high temperatures, soil salinity, and heavy metal toxicity, are essential [4,5].

Research on combining multiple agronomic and environmental factors, such as fertilizer supply and water management, has led to the development of comprehensive strategies to enhance crop resilience and productivity. Precision irrigation, nitrogen cycling, and soil remediation techniques are crucial for sustainably enhancing productivity [6]. In particular, researchers seeking to create crops that can flourish in harsh environmental conditions like drought, high salinity, and nutrient deficiencies have made enhancing stress resilience in crops through integrated nutrient management and eco-friendly practices a top priority [7,8].

Furthermore, an increasing amount of research highlights the significance of soil bacteria in sustainable farming methods. Research shows that interactions between soil bacteria and plants can improve plant stress tolerance, encourage nutrient uptake, and have a significant impact on the nutrient cycle [9]. This demonstrates how soil-based biological interventions can support conventional nutrient management techniques, lowering the requirement for chemical fertilizers while enhancing soil fertility and plant health.

Lastly, the necessity of implementing resilient agriculture practices has increased due to climate change, which has increased the frequency of extreme weather occurrences. Research on more climate-resilient plant species and adaptive management techniques has been prompted by the effects of droughts, flooding, and rising temperatures on crop yields [10]. We can develop strategies that will guarantee the long-term sustainability of plant production systems by combining climate-smart farming practices with sustainable nutrient management.

## 2. Advances in Sustainable Approaches to Plant Nutrient and Environment Management

This section provides an overview of the articles included in this SI, highlighting how their findings collectively advance research on sustainable nutrient and environmental management in plant production.

Wetlands in semi-arid regions struggle with issues such as salt, alkalinity, and water scarcity. In this study [11], the nitrogen (N) and phosphorus (P) stoichiometry of *Bolboschoenus planiculmis*, a significant plant in China’s Songnen Plain wetlands, was investigated in this work. Although it changed the plant’s N and P content and use techniques, agricultural drainage water was found to reduce salinization and aridification. The work provides insights into how sustainable water management techniques can enhance vegetation health in degraded wetlands by highlighting the critical influence of water variables, such as temperature and nitrate content, on nutrient dynamics.

One of the principal abiotic stresses limiting soybean productivity is soil salinity [12]. The function of poly (acrylic) acid-coated Mn_3_O_4_ (PMO) nanoparticles in improving soybean germination under salt stress was investigated in this work. The findings demonstrated that PMO treatment increased seedling growth and germination rates, decreased reactive oxygen species, and strengthened antioxidant defense. This research provides fresh insights into how nanoparticles can enhance plant stress tolerance, offering a viable option for crops grown in saline conditions.

Integrating Chinese milk vetch (CMV) and rice straw (RS) with reduced chemical fertilizers (CFs) is a promising approach to reduce reliance on synthetic fertilizers without compromising crop yields [13]. When compared to standard fertilization, the MSF80 treatment (80% CF plus RS and CMV) increased rice yields and improved nutrient usage efficiency during four years. To maintain sustainable rice production methods, promote long-term soil health, and reduce chemical fertilizer inputs, the study recommends using organic additions.

The effects of various ammonium and nitrate nitrogen ratios on nitrogen uptake, gene expression, and rice yield were investigated thoroughly [14]. When compared to using ammonium alone, the results showed that a 75:25 ammonium-nitrate mixture considerably enhanced root biomass, tiller number, and yield. Furthermore, the combination increased the expression of genes associated with nitrogen metabolism, thereby improving the efficiency of nitrogen utilization. These results suggest that increasing rice yield and reducing nutrient inefficiencies through nitrogen form optimization can support sustainable farming practices.

The findings of Muhammad et al. [15] revealed the long-term effects of nitrogen fertilizer and microbial biochar on reducing salt stress in paddy soil. Nitrogen fertilizer and microbial biochar, especially bacterial biochar (BB), significantly improved soil characteristics, reduced soil salinity, and enhanced rice growth, particularly in salt-tolerant varieties. The study demonstrates that microbial biochar has the potential to be a practical and sustainable approach for mitigating salt stress in rice farming.

According to Ashraf et al. [16], rice development, yield, and grain quality are significantly hampered by lead (Pb) and cadmium (Cd) poisoning. The function of exogenous gamma-aminobutyric acid (GABA) in reducing Pb and Cd toxicity in the aromatic rice, *Guixiangzhan,* was evaluated in this study. The findings showed that GABA significantly increased antioxidant, carotenoid, and chlorophyll levels, while reducing oxidative stress and enhancing grain production. Exogenous GABA appears to be a promising method for reducing Pb and Cd toxicity in contaminated soils and increasing rice yield, according to this study.

The findings of Qi et al. [16] examined the physiological and biochemical reactions of heat-tolerant and heat-sensitive rice genotypes to high-temperature stress, as climate change is exacerbating heat stress. The heat-sensitive genotype, HTS-5, experienced oxidative damage, whereas the heat-tolerant genotype, HTR-1, demonstrated improved tolerance through increased antioxidant activity and higher anther dehiscence. The study highlights HTR-1’s potential as a tool for producing heat-tolerant rice cultivars, thereby increasing the resilience of rice production to high temperatures.

Litchi’s poor fruit-setting rate makes production difficult [17]. Auxin’s function in litchi floral differentiation was investigated in this study, which discovered that male flowers had higher auxin levels than female flowers. During flower bud development, auxin-related genes showed differential expression, according to transcriptome analysis. These discoveries deepen our knowledge of the processes behind floral sexual differentiation in litchi, laying the groundwork for methods to control flowering and boost fruit yield in this important crop.

## 3. Conclusions

To summarize, this Special Issue highlights key developments in the sustainable management of plant nutrients and the environment. The studies included in this compilation underline the importance of creative methods in increasing plant yields while minimizing negative environmental impacts. The research provides essential insights for developing more sustainable agricultural techniques, ranging from methods to mitigate the effects of heavy metal toxicity in rice to strategies to optimize nitrogen fertilization and enhance crop resistance to salt stress. In my capacity as Guest Editor, I would like to express my sincere gratitude to all of the writers for their exceptional work and to the reviewers for their thorough assessments, which guaranteed the scientific integrity of the pieces published in this SI. We aim that the results reported here will stimulate further investigation and offer useful advice for the creation of plant production systems that are more robust and sustainable.
